# The Influence of Atopic Dermatitis on Health-Related Quality of Life in Bangladesh

**DOI:** 10.3390/ijerph182111593

**Published:** 2021-11-04

**Authors:** Abir Nagata, Taheruzzaman Kazi, Zubaida Akter, Fariha Afrin Nody, Mohammad Shahriar Khan, Abu Saleh Muhammad Shahriar, Md Sayeedul Islam, Takatoshi Nakagawa, Shigeki Inui

**Affiliations:** 1 Department of Regenerative Dermatology, Graduate School of Medicine, Osaka University, Suita 565-0871, Japan; tanjim@r-derma.med.osaka-u.ac.jp (T.K.); tnakagawa@r-derma.med.osaka-u.ac.jp (T.N.); inui@r-derma.med.osaka-u.ac.jp (S.I.); 2 Department of Dermatology and Venereology, Shaheed Suhrawardy Medical College Hospital, Dhaka 1207, Bangladesh; akhterzubaida@yahoo.com; 3 Ibrahim Cardiac Hospital and Research Institute, Dhaka 1000, Bangladesh; fariha.nody21@gmail.com; 4 International Platform for Dryland Research and Education, Tottori University, Tottori 680-0001, Japan; shahriarkhan0015@gmail.com; 5 Department of Periodontology and Oral Pathology, University Dental College, Dhaka 1217, Bangladesh; dr.shahriarshawon@gmail.com; 6 Department of Biological Sciences, Graduate School of Science, Osaka University, Toyonaka 560-0043, Japan; islam@bio.sci.osaka-u.ac.jp

**Keywords:** atopic dermatitis, disease severity, Dermatology Life Quality Index, Eczema Area and Severity Index, health-related quality of life

## Abstract

Atopic dermatitis (AD) is the foremost non-fatal skin-related disease that affects all age groups. Despite the growing prevalence of AD in low- and middle-income countries, its physiological consequences remain overlooked in countries like Bangladesh. Therefore, we aim to assess and characterize the influence of AD on the health-related quality of life (HRQoL) in Bangladeshi patients. A cross-sectional study comprising 184 eligible adults (83 men and 101 women; mean age, 33.46 ± 15.44 years) was conducted at the dermatology outpatient department of Shaheed Suhrawardy Medical College Hospital (a tertiary hospital in Dhaka, Bangladesh). AD was determined using the UK Working Party criteria. A structured questionnaire, Eczema Area and Severity Index (EASI), and Dermatology Life Quality Index (DLQI) were administered to obtain information on patient characteristics, AD severity, and HRQoL. The mean DLQI score for the entire sample was 11.29 ± 5.27 (range, 1–26), and 51.60% reported the disease greatly affected their lives. Bivariate analysis revealed significant differences in self-rated health measures of DLQI scores in terms of self-reported AD severity, overall health, and the EASI. In multivariable regression models adjusted for patient characteristics, the self-perceived severe AD group reported significantly higher DLQI scores (coefficient = 2.72; 95% confidence interval (CI) = 0.38–5.05; *p* = 0.022) than the mild group. Concurrently, we observed a substantial increase in the DLQI scores among patients with moderate and severe EASI scores (coefficient = 1.96, 95% CI = 0.08–3.92, *p* < 0.05 and coefficient = 4.35, 95% CI = 1.98–6.72, *p* < 0.001, respectively) than in those with mild EASI scores, suggesting that HRQoL was markedly influenced by greater AD severity. These findings highlight the need for a more patient-centric approach to the management of AD in order to alleviate patient suffering and, thereby, improve HRQoL.

## 1. Introduction

Atopic dermatitis (AD) is a chronic, relapsing inflammatory skin disorder characterized by frequent exacerbations of pruritic and eczematous lesions [1]. The pathogenesis of AD is complex and involves genetic susceptibility, abnormal skin barrier function, dysfunctional cell-mediated immunity, and environmental and lifestyle factors [1,2]. AD affects people of all ages, but it typically manifests in early infancy or childhood, persists into adulthood, and chronically affects patients throughout their lives [1,3]. However, new-onset AD can also develop in adults, and, hence, studies on adult-onset AD have increased [3,4]. This heterogeneous skin disorder with wide-ranging clinical manifestations, including inflammatory infiltration, intense pruritus, and skin pain, can impose a profound burden on patients [4,5]. According to the Global Burden of Disease project, AD contributes to the highest disease burden among all skin disorders, as measured using disability-adjusted life years, and ranks 15th among all non-fatal diseases [6]. Moreover, AD may lead to psychological stress due to stigmatization, impaired physical and social functioning, and sleep disturbance, thereby considerably undermining different aspects of a patient’s health-related quality of life (HRQoL) [7,8].

HRQoL is a multidimensional concept that reflects an individual’s subjective feeling of health; it has become an essential indicator for patient health in dermatology as skin diseases are usually long-lasting and have a marked impact on a patient’s life [8,9]. Many studies have highlighted the relevance of HRQoL in adults with AD [5,7,8]. Compared to the general population and those with other chronic diseases such as diabetes mellitus and heart disease, adult patients with AD show a decrease in HRQoL [7]. Evidence also suggests that adults with AD experience embarrassment, self-consciousness, and interference with personal and social relationships, emotional state, work performance, and daily activities [7]. Therefore, dermatologists are strongly encouraged to examine patients’ psychological aspects, of which HRQoL is a good indicator. In patients with AD, HRQoL is affected by greater AD severity (especially when the affected body surface area is larger), pruritus severity, skin pain, frequency of sleep disturbance, and skin dryness [7,8,10]. Consequently, the quantification of HRQoL related to AD severity is being increasingly emphasized since this assessment provides additional information for patient management.

AD has been linked to a significant disease burden in Asian countries and has an impact on the quality of life (QoL) of the patients and their families [11]. Moreover, in recent decades, the prevalence of AD in Asia, including low- and middle-income countries (LMICs), has seen an increase, probably because of increasing urbanization, westernized lifestyles, and improved living standards [11,12]. Notably, owing to the higher rates of disease onset during adulthood, the prevalence of AD in adults seems to be greater in some Asian populations than in other populations [12]. However, age of onset, disease severity, and genetic susceptibility may differ between and within countries as well as between rural and urban areas [13]. Therefore, understanding the psychological influence of AD and characterizing it in each population is imperative to alleviate patient suffering and optimize care. Moreover, as AD is incurable, patient satisfaction and HRQoL improvement in accordance with symptom reduction have become increasingly vital in clinical decision-making [8].

While the psychological burden of AD is well documented in high-income countries (HICs), including those in Asia, its effects have rarely been investigated in LMICs. In Bangladesh, AD is one of the most common skin diseases, and its prevalence has been increasing recently [14,15]. However, the psychological aspects of AD in Bangladeshi adults have not yet been evaluated. Hence, the objective of this study is to evaluate HRQoL and characterize factors that influence HRQoL among Bangladeshi adults with AD by using the Bangla version of the Dermatology Life Quality Index (DLQI). The DLQI has been well-validated and widely used to measure subjective impairment in several dermatologic conditions, including AD [16]. Additionally, we examined the reliability of the Bangla version of the DLQI.

## 2. Materials and Methods

### 2.1. Study Design and Participants

A non-interventional, cross-sectional study was performed to assess HRQoL and associated factors in patients with AD. Participants for this study were recruited consecutively between January and April 2021 from among the patients who visited the dermatology outpatient department of the Shaheed Suhrawardy Medical College Hospital (a tertiary hospital in Dhaka, Bangladesh). The inclusion criteria were as follows: (a) patients with AD, (b) aged 16 years or older, (c) able to communicate in Bangla language, and (d) voluntarily agreeing to participate after providing written informed consent. Patients with any of the following conditions were excluded: (a) pregnant or lactating women, (b) patients with mental illness, and (c) patients having difficulty completing the surveys. We adopted the UK Working Party (UKWP) criteria to identify patients with AD [17]. This included all characteristics of the UKWP criteria, including skin itchiness during the last 12 months and three or more of the following: history of (1) skin crease involvement, (2) asthma or hay fever, (3) general dry skin during the past year, (4) visible flexural eczema, or (5) onset before the age of 2 years. Patients who met the inclusion criteria were invited to participate in the study. Patients with active dermatological diseases other than AD were excluded from the final analysis. Throughout the study, patient identification and disease severity examination were performed by one dermatologist, and this was followed by an interview by a trained enumerator.

### 2.2. Measurement Tools

#### 2.2.1. Measurement of AD Severity

Disease severity was assessed using the Eczema Area and Severity Index (EASI), which is a broadly used and validated tool for measuring the severity of AD [18]. The EASI was applied to structure the observations because it is simple, easy to understand, and can be used by practitioners to normalize the baseline evaluation of the condition and track changes over time. The EASI encompasses an assessment of disease extent on a scale of 0–6 in four affected body parts (head/neck, trunk, and upper and lower extremities) and an evaluation of erythema, infiltration/papulation, excoriation, and lichenification, each scored on a scale of 0–3. The sum of these scores yields the EASI, which ranges from 0 to 72. A higher EASI score indicates extensive and severe disease conditions. In our study, we divided patients with AD into groups of equal percentiles based on their EASI scores: mild (<9), moderate (9–15), and severe (>15), as no cutoff values had been proposed for this measure.

#### 2.2.2. Measurement for HRQoL

A 1-week-recall, Bangla edition of the DLQI questionnaire was administered as a self-assessment of the patients’ HRQoL. The DLQI is a dermatology-specific questionnaire designed for adults (i.e., patients aged > 16 years) to evaluate any skin disorder that affects different aspects of their lives over the previous week [16,19,20]. The DLQI consists of 10 items evaluating disease impact on key aspects of patient lives and is distributed across six domains related to symptoms and feelings (Questions 1 and 2), daily activities (3 and 4), leisure (5 and 6), work and study (7), personal relationships (8 and 9), and treatment (10), as well as an overall DLQI score. Each DLQI question has four response categories (“not at all”, “a little”, “a lot”, and “very much”) that are scored on a 0–3 Likert scale. The overall DLQI is the total score of all questions and ranges from 0 to 30, with a higher score indicating increased QoL impairment. The overall DLQI score can be classified into five groups according to the influence on the patients’ HRQoL: no effect (0–1), a small effect (2–5), a moderate effect (6–10), a very large effect (11–20), and an extremely large effect (21–30).

#### 2.2.3. Self-Assessed AD Severity, Overall Health, and Other Study Variables

To obtain the patients’ subjective appraisal of their condition, we chose a combination of three questions dealing with previously validated “self-reported global AD severity” (how would you describe your AD: mild, moderate, or severe) [21] and “self-assessment of overall health” (how would you describe your overall health: poor, fair, good, or excellent). The demographic data collected included age, sex (male/female), educational level (≤primary, secondary, and ≥tertiary), marital status (single/married), and residence (rural/urban). We also collected information on current smoking (yes/no), family history of AD (yes/no), and allergic complications (yes/no). All the questionnaires (except for the DLQI questionnaire) were first prepared in English and then translated into Bangla.

### 2.3. Data Analysis

The sample size for this study was determined using G Power 3.1 software [22] based on a significance level of 0.05, a power of 0.95, and a medium-size effect of 0.15. The analysis revealed that 178 participants were required for the analysis. Patient characteristics were expressed as frequencies, percentages, means, and standard deviations. First, the internal consistency reliability of the DLQI items was examined using Cronbach’s α coefficients and item-total correlations. Cronbach’s α, which is a highly relevant method to measure the reliability of a scale containing multiple items, was defined for the overall instrument [16]. Cronbach’s α coefficient value of not less than 0.7 is generally considered sufficient to demonstrate internal consistency [16,23]. For item-total correlation, a value of >0.3 is considered to indicate that an item is connected to the overall scale [23]. Bivariate and multivariable analyses were performed to identify the factors influencing HRQoL among patients with AD. First, a bivariate analysis was conducted to explore the differences across the patients’ general characteristics using domain-specific DLQI and overall DLQI scores. Non-parametric approaches, the Mann–Whitney U-test and the Kruskal–Wallis test, were used because the variables had a non-normal distribution according to the Shapiro–Wilk test. Subsequently, posthoc F tests were performed to detect any potential differences between the groups.

Second, multiple linear regression analyses were adopted to determine the association between illustrative variables: domain-specific DLQI, overall DLQI, and patient characteristics. Individual variables that showed statistical significance (*p* < 0.05) at the bivariate level were included in the regression model. Domain-specific DLQI (symptoms and feelings, daily activities, leisure, work and school, personal relationships, and treatment) and overall DLQI scores were used separately as dependent variables in the model, and the potential confounders adjusted for were marital status, family history of AD, self-reported global AD severity, overall health ratings, and EASI scores. We included the variance inflation factor (VIF) in each regression model to check for multicollinearity; however, none of the variables exceeded a VIF > 2.0. The beta coefficient was also determined to evaluate the strength of the association with a 95% confidence interval (CI). The significance level was set to *p* < 0.05. Statistical analyses were performed using IBM SPSS Statistics for Windows/Macintosh, Version 25.0 (IBM Corp., Armonk, NY, USA).

## 3. Results

### 3.1. Patient Characteristics

**Figure 1:**
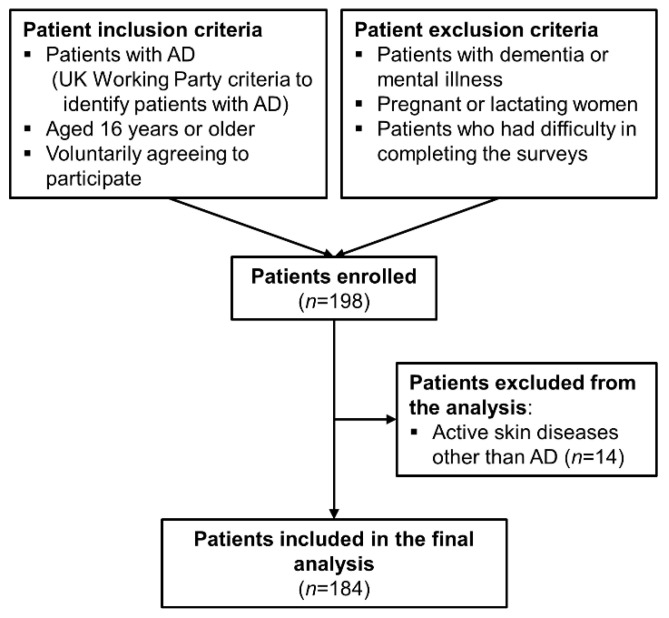
Overview of the patient enrollment process.

Of the 198 patients enrolled, 14 were excluded because they had active skin diseases other than AD, thus leaving 184 to be included in the final analysis (Figure 1). Table 1 presents the descriptive characteristics of the patients. The participants included 83 men (45.10%) and 101 women (54.90%) with a mean age (±standard deviation) of 33.46 ± 15.44 years (range, 16–70 years), and the highest proportion of patients was in the age group below 30 years (54.30%). In total, 52.20% of patients had completed tertiary education, and 57.60% were married. Moreover, 97 (52.70%) patients reported having a family history of AD, and 98 (53.30%) had allergy complications. In terms of self-reported global AD severity, 21 (11.40%) patients reported having mild AD, 51 (27.70%) as having moderate AD, and 112 (60.90%) as having severe AD. The overall health rating of the patients was poor in 37 (20.10%), fair in 35 (19.00%), good in 98 (53.30%), and excellent in 14 (7.60%). The distribution of disease severity was expressed on the basis of the cutoff value of the EASI score. The severity of AD was mild, moderate, and severe in 33 (17.90%), 97 (52.70%), and 54 (29.40%) patients, respectively.

### 3.2. Descriptive Summary of DLQI Scale Scores, Internal Consistency, and Overall HRQoL

The descriptive summary and internal consistency reliability of the DLQI items are presented in Table 2. The internal reliability coefficient (measured using Cronbach’s α) for the DLQI was 0.75. As shown in Table 2, Cronbach’s α ranged between 0.71 and 0.76 for all 10 items of the DLQI questionnaire, indicating good internal consistency and no redundant items. The item-total correlation for all the 10 items exhibited good correlation (ranging between 0.23 and 0.63), except for Item 9. The mean overall DLQI score for the whole sample was 11.29 ± 5.27 (range, 1–26). Of the 10 DLQI scales, the highest score was recorded for Question 1 (itchy and painful; 2.15 ± 1.08), followed by Question 2 (embarrassment; 1.89 ± 1.06). Figure S1 represents the distribution of patient responses on the DLQI items. Based on the overall DLQI score classification, only 2.7% of patients reported AD having “no effect on their life”, while 28.80% and 51.60% of patients reported AD having “mild” and “very large effect on their life”, respectively (Table 3).

### 3.3. HRQoL According to Patient Demographic Characteristics

The association between domain-specific and overall DLQI and patient demographic characteristics at the bivariate level is shown in Table 4. Significant differences in self-rated health measures of domain-specific DLQI and overall DLQI scores were found in relation to marital status and family history of AD. Scores of “daily activities”, “leisure”, “personal relationship”, and overall DLQI were significantly higher in the married group than in the single group. Patients who had a family history of AD reported higher scores in “symptoms and feelings”, “daily activities”, “leisure”, “personal relationship”, and overall DLQI.

### 3.4. HRQoL According to Patient Self-Perceived AD Severity, Overall Health Rating, and the EASI

**Figure 2:**
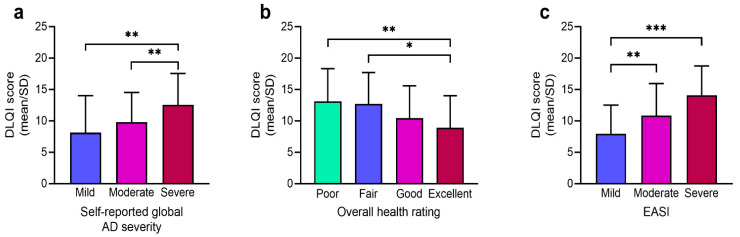
Bivariate association between the overall DLQI score (expressed as mean/SD) and subgroups of (**a**) self-reported global AD severity, (**b**) overall health rating, and (**c**) the EASI. EASI categorized as: scores <9 (mild), 9–15 (moderate), and >15 (severe). DLQI: Dermatology Life Quality Index; EASI: Eczema Area and Severity Index; AD: atopic dermatitis. *** *p* < 0.001; ** *p* < 0.01; * *p* < 0.05. note: A higher DLQI value indicates a poorer HRQoL.

Bivariate association between the patients’ HRQoL and self-perceived AD severity, overall health rating, and the EASI are shown in Table 5 and Figure 2. A significant difference was observed in “symptoms and feelings”, “daily activities”, and “personal relationship” in relation to self-perceived AD severity and the EASI. Likewise, the overall DLQI scores were significantly higher (*p* < 0.01) in the self-perceived moderate and severe AD groups than in the mild AD group (Figure 2a). Those with poor and fair self-perceived overall health rated significantly higher DLQI scores (*p* < 0.01 and *p* < 0.05, respectively) than did those with excellent overall health (Figure 2b). We also observed a significant increase in the overall DLQI score in the moderate and severe AD groups (*p* < 0.001 and *p* < 0.01, respectively) than in the mild AD group (Figure 2c).

### 3.5. Factors Influencing HRQoL

The models used to estimate the factors influencing domain-specific DLQI are presented in Table 6. “Leisure”-related QoL was significantly associated with a “family history of AD” (*p* < 0.01). Patients with “self-perceived severe AD” were more likely to have problems with QoL related to “symptoms and feelings” (*p* < 0.01) and “work and school” (*p* < 0.05). However, we found that all the domain-specific DLQI scales (except “work and school”) were significantly associated with severe EASI scores, indicating that severe EASI groups are more likely to experience problems with QoL associated with “symptoms and feelings”, “personal relationships”, and “treatment”. In Table 7, the regression model revealed that the patients’ HRQoL was considerably influenced by a “family history of AD” (coefficient = 1.49; 95% CI = 0.02–2.97; *p* < 0.047). Then again, in “self-reported global AD severity”, the severe AD group showed a significant association with the overall DLQI (coefficient = 2.72; 95% CI = 0.38–5.05; *p* < 0.05), indicating poorer HRQoL than in the mild AD group. Concurrently, a significant increase in DLQI scores was observed among patients with moderate and severe EASI scores (coefficient = 1.96, 95% CI = 0.08–3.92, *p* < 0.05 and coefficient = 4.35, 95% CI = 1.98–6.72, *p* < 0.001, respectively) than among those with mild EASI scores, suggesting a marked impairment in HRQoL with disease severity. However, no significant association with HRQoL was observed for sociodemographic parameters and overall health ratings.

## 4. Discussion

To our knowledge, this is the first study in Bangladesh to assess HRQoL and associated factors in adults with AD, including a test to investigate the internal consistency reliability of the DLQI items. Internal consistency reliability measures the homogeneity of multiple items and is considered a test of the reproducibility of an instrument [16]. For instance, when a patient’s health status remains stable over time, the results of the instruments should also remain stable [9]. In our study, we found that the internal consistency reliability of the DLQI items was good, as judged using Cronbach’s α coefficients and item-total correlations. These findings are similar to those obtained in the original validation study and studies using the DLQI in other languages [16,19]. Therefore, we suggest that the Bangla version of the DLQI may serve as a potential instrument for AD-related health status measurement in Bangladesh and may contribute to cross-cultural comparison. In addition, the DLQI offers advantages such as brevity, low burden to participants and data collectors, and ease of interpretation. Furthermore, the implementation of the DLQI would help clinicians address specific complications in the patients and categorize which aspects of the patients’ lives are most severely affected [20].

We observed that poorer HRQoL was associated with the DLQI scores; half of the participants reported having very large effects on their lives, and these outcomes were mostly influenced by greater AD severity. Among the participants, the mean DLQI score was 11.29, which was relatively higher than that reported in several previous studies in HICs [21,24,25,26]. Notably, a cross-sectional population-based study of 602 US adults reported a mean DLQI score of 4.9 [26]. In contrast, Vakharia et al. found a mean DLQI score of 10.7 among 265 US adults, while Holm et al. and Sanchez-Perez et al. found mean DLQI scores of 9.79 and 7.8, respectively, among Danish and Spanish patients [21,24,25]. However, a recent review of 27 studies in Asia found that the DLQI values ranged between 4.8 and 12.0 [27]. Similarly, another review of 32 studies across the US, Europe, and some Asian countries suggested that the mean DLQI scores varied between 4.9 and 20 [7]. The same review addressed the difficulty in interpreting the overall score because of its variability across studies and the lack of formal reference values or population normal scores for formal comparisons. Therefore, more in-depth qualitative research is needed to confirm these findings.

A substantial number of reports support the role of disease severity as an important risk factor for HRQoL impairment in patients with AD [5,6,7,8,27]. In our study, we examined the influence of AD on HRQoL in association with disease severity, measured using both the clinical objective scoring system EASI and self-rated global AD severity. Both bivariate and multivariable regression analyses showed that patients with moderate and severe AD reported worse HRQoL than those with mild AD. This is consistent with the findings of previous reports that support the hypothesis that HRQoL decreases as disease severity increases [28,29]. In a systematic review of 15 studies in Asia, 14 studies showed a significant association between disease severity and HRQoL impairment [27]. In another review, 19 out of 20 studies showed that increased disease severity was significantly associated with poorer HRQoL [7]. The effects of AD on impaired HRQoL in our study seem mostly related to itchiness and embarrassment as these parameters were the most commonly recorded in the DLQI. Notably, chronic pruritus and eczematous lesions are the most common symptoms in AD that may affect the patient’s mood and lead to embarrassment, thereby impacting the patient’s psychological well-being. In contrast, greater severity was associated with greater impairment in several dimensions of the DLQI, namely, symptoms and feelings, daily activities, leisure, personal relationships, and treatment, which is consistent with the findings of previous research [29,30]. Evidence also suggests that personal relationships were the least affected dimension of HRQoL [7]; however, we found that patients aged between 30 and 50 years experienced more problems in personal relationships than did those in other age groups. Moreover, we noticed that having a family history of AD negatively influenced HRQoL, especially leisure-related QoL. As AD is a restrictive disease that interferes with daily activities, social and interpersonal relationships would obviously be affected in patients. Furthermore, in LMICs, limited access to treatment, disease management, lack of formal diagnosis, and low socioeconomic status, which possibly result in poor follow-up in clinics, may contribute to impaired HRQoL in patients with AD [11,12,15].

Several studies showed no clear association between the patients’ demographic characteristics and HRQoL impairment [27,31]. Our analysis also did not reveal such an association between demographic characteristics and HRQoL. However, Holm et al. found more impaired HRQoL in female patients than in male patients [24]. Therefore, further studies are required to clarify these relationships. Self-perceived poor overall health was associated with AD in a study by Silverberg et al. [26]. In our study, the bivariate analysis showed an association between self-perceived overall health and HRQoL in AD; however, in the regression model adjusted for potential confounders, this association was diminished. One explanation for this observation may be that the participants in our study visited a tertiary hospital because of the main complaint of skin problems. Therefore, disease severity seems to be the main factor that influences HRQoL in Bangladeshi adult patients with AD.

Several limitations of this study warrant consideration. First, the outcomes of a cross-sectional study cannot be taken as evidence of causal relationships. Second, the single-institution study design limits the generalizability of the results. Third, we did not collect information on the age of AD onset, current treatment therapy, sleep disorders, and HRQoL using generic questionnaires. Lastly, we did not include healthy subjects as a control group; therefore, our observations need further validation. Nevertheless, to our knowledge, this study is the first of its kind in Bangladesh to document HRQoL among adults with AD. In addition, we used the objective scoring system EASI to evaluate disease severity. The EASI allows for individual items to be measured, either separately or in combination, thereby providing a more comprehensive evaluation and acting as a reference for clinical disease severity in patients. Further research on a larger patient cohort, including more medical information and utilizing both generic and dermatology-specific QoL questionnaires, is required to better quantify the effects of AD and ensure effective treatment.

## 5. Conclusions

In conclusion, this study demonstrates that Bangladeshi adults with AD experience substantially poorer HRQoL and that the degree of impairment is markedly influenced by greater AD severity. Most of the patients experienced the effects of “symptoms and feelings”, “daily activities”, “leisure”, “personal relationships”, and “treatment” on their QoL. In addition, employing the DLQI instrument seemed beneficial in identifying vulnerable patients for whom appropriate treatment strategies have to be designed and for whom the therapeutic response has to be measured. Therefore, we recommend that dermatologists be aware of the clinical characteristics and negative psychosocial impacts of AD on the HRQoL of patients with this condition.

## Figures and Tables

**Table 1:** Demographic and clinical characteristics of the patients with AD (*n* = 184).

Variable		*n* (%)
Sex	Male	83 (45.10)
	Female	101 (54.90)
Age (years)	<30	100 (54.30)
	30–50	55 (29.90)
	>50	29 (15.80)
Level of education	≤Primary	64 (34.80)
	Secondary	24 (13.00)
	≥Tertiary	96 (52.20)
Marital status	Single	78 (42.40)
	Married	106 (57.60)
Residence	Rural	40 (21.70)
	Urban	144 (78.30)
Smoking	Yes	20 (10.90)
	No	164 (89.10)
Family history of AD	Yes	97 (52.70)
	No	87 (47.30)
Allergic complications	Yes	98 (53.30)
	No	86 (46.70)
Self-reported global AD severity	Mild	21 (11.40)
	Moderate	51 (27.70)
	Severe	112 (60.90)
Overall health rating	Poor	37 (20.10)
	Fair	35 (19.00)
	Good	98 (53.30)
	Excellent	14 (7.60)
EASI	Mild (<9)	33 (17.90)
	Moderate (9–15)	97 (52.70)
	Severe (>16)	54 (29.40)

AD: atopic dermatitis; EASI: Eczema Area and Severity Index.

**Table 2:** Summary descriptive statistics and internal consistency reliability of the DLQI items.

DLQI Item	Mean (SD)	Reliability	
		Cronbach’s α coefficient	Item-total correlation
Itchy, painful	2.15 (1.08)	0.737	0.437
Embarrassed	1.89 (1.06)	0.718	0.551
Interfered with shopping, housework	0.98 (1.04)	0.722	0.530
Influenced clothes	1.07 (1.11)	0.744	0.395
Affected social activities	0.67 (0.85)	0.711	0.636
Affected sports	0.47 (0.78)	0.749	0.340
Prevented work	1.11 (0.75)	0.753	0.304
Problem with friends	0.74 (0.87)	0.734	0.461
Sexual difficulties	0.47 (0.73)	0.760	0.237
Treatment problematic	1.75 (0.97)	0.752	0.326
Total DLQI score	11.29 (5.27)		

Values of DLQI items expressed as mean ± SD. DLQI: Dermatology Life Quality Index; SD: standard deviation.

**Table 3:** Distribution of patients with AD based on the DLQI score ranges and their significance on the patients’ lives.

DLQI Score Range	Meaning of Scores	*n*	%
0–1	No effect at all on patient’s life	5	2.70
2–5	Small effect on patient’s life	26	14.20
6–10	Moderate effect on patient’s life	53	28.80
11–20	Very large effect on patient’s life	95	51.60
21–30	Extremely large effect on patient’s life	5	2.70

AD: atopic dermatitis; DLQI: Dermatology Life Quality Index.

**Table 4:** Bivariate association between the patients’ demographic characteristics and domain-specific and overall DLQI scores.

Variable		Symptoms and Feelings	Daily Activities	Leisure	Work and School	Personal Relationships	Treatment	DLQI
		Mean ± SD	Mean ± SD	Mean ± SD	Mean ± SD	Mean ± SD	Mean ± SD	Mean ± SD
Sex	Male	3.93 ± 1.79	1.84 ± 1.65	1.31 ± 1.63	1.17 ± 0.71	1.19 ± 1.24	1.66 ± 0.94	11.11 ± 5.50
	Female	4.13 ± 1.81	2.22 ± 1.70	0.99 ± 1.10	1.06 ± 0.78	1.23 ± 1.22	1.82 ± 1.00	11.45 ± 5.09
	*p*	0.367	0.098	0.460	0.277	0.745	0.310	0.667
Age	<30	3.96 ± 1.84	1.84 ± 1.69	0.98 ± 1.31	1.10 ± 0.77	0.96 ± 1.08	1.67 ± 0.98	10.51 ± 5.12
	30–50	3.98 ± 1.66	2.25 ± 1.68	1.27 ± 1.45	1.16 ± 0.71	1.65 ± 1.41	1.93 ± 1.01	12.25 ± 5.60
	>50	4.41 ± 1.93	2.38 ± 1.63	1.41 ± 1.37	1.03 ± 0.77	1.24 ± 1.12	1.69 ± 0.85	12.17 ± 4.83
	*p*	0.371	0.121	0.096	0.718	0.051	0.256	0.088
Level of education	≤Primary	4.28 ± 1.74	2.28 ± 1.65	1.27 ± 1.46	1.20 ± 0.76	1.31 ± 1.15	1.86 ± 1.00	12.20 ± 5.02
	Secondary	3.46 ± 2.00	1.67 ± 1.65	1.17 ± 1.68	1.00 ± 0.59	1.13 ± 1.32	1.92 ± 0.92	10.33 ± 6.02
	≥Tertiary	4.02 ± 1.77	1.99 ± 1.70	1.04 ± 1.23	1.07 ± 0.78	1.17 ± 1.26	1.64 ± 0.96	10.93 ± 5.20
	*p*	0.196	0.233	0.611	0.450	0.456	0.217	0.206
Marital status	Single	3.83 ± 1.81	1.72 ± 1.69	0.91 ± 1.34	1.09 ± 0.79	0.88 ± 1.03	1.59 ± 0.98	10.03 ± 5.08
	Married	4.19 ± 1.78	2.29 ± 1.64	1.30 ± 1.38	1.12 ± 0.72	1.45 ± 1.31	1.87 ± 0.95	12.23 ± 5.23
	*p*	0.198	**0.010**	**0.013**	0.969	**0.002**	0.065	**0.005**
Residence	Rural	4.03 ± 1.68	2.13 ± 1.68	1.33 ± 1.43	1.08 ± 0.65	1.33 ± 1.20	1.75 ± 0.98	11.63 ± 5.11
	Urban	4.04 ± 1.83	2.03 ± 1.69	1.08 ± 1.35	1.12 ± 0.78	1.18 ± 1.23	1.75 ± 0.97	11.20 ± 5.32
	*p*	0.821	0.750	0.202	0.850	0.418	0.992	0.654
Smoking	Yes	4.20 ± 1.67	2.00 ± 1.86	1.45 ± 1.66	1.25 ± 0.63	1.35 ± 1.34	1.90 ± 0.78	12.15 ± 5.86
	No	4.02 ± 1.82	2.05 ± 1.67	1.10 ± 1.33	1.09 ± 0.76	1.20 ± 1.21	1.73 ± 0.99	11.19 ± 5.20
	*p*	0.778	0.758	0.369	0.331	0.633	0.512	0.443
Family history of AD	Yes	4.46 ± 1.58	2.35 ± 1.68	1.43 ± 1.36	1.16 ± 0.73	1.43 ± 1.31	1.79 ± 0.91	12.64 ± 4.82
	No	3.56 ± 1.92	1.71 ± 1.63	0.80 ± 1.31	1.05 ± 0.77	0.97 ± 1.08	1.70 ± 1.04	9.79 ± 5.36
	*p*	**0.003**	**0.007**	**0.002**	0.253	**0.012**	0.539	**<0.001**
Allergic complications	Yes	4.02 ± 1.83	2.06 ± 1.53	1.28 ± 1.44	1.00 ± 0.70	1.18 ± 1.21	1.68 ± 0.98	11.22 ± 5.15
	No	4.06 ± 1.77	2.03 ± 1.85	0.98 ± 1.28	1.23 ± 0.79	1.24 ± 1.25	1.83 ± 0.97	11.37 ± 5.43
	*p*	0.923	0.571	0.103	0.052	0.745	0.303	0.850

Values of domain-specific and overall DLQI expressed as mean ± SD. DLQI: Dermatology Life Quality Index; SD: standard deviation; AD: atopic dermatitis; EASI: Eczema Area and Severity Index; *p*-value calculated using the Mann–Whitney U-test or Kruskal–Wallis test; values in bold are significant.

**Table 5:** Bivariate association between domain-specific, overall DLQI scores and self-perceived AD severity, overall health rating, and the EASI.

Variable		Symptoms and Feelings	Daily Activities	Leisure	Work and School	Personal Relationships	Treatment	DLQI
Self-reported global AD severity	Mild	2.90 ± 2.02	1.38 ± 1.62	0.90 ± 1.44	0.81 ± 0.87	0.81 ± 0.92	1.33 ± 1.01	8.14 ± 5.87
	Moderate	3.59 ± 1.81	1.63 ± 1.45	0.98 ± 0.96	0.98 ± 0.73	0.94 ± 1.17	1.67 ± 0.86	9.75 ± 4.75
	Severe	4.46 ± 1.62	2.37 ± 1.72	1.25 ± 1.51	1.22 ± 0.71	1.41 ± 1.27	1.87 ± 1.00	12.57 ± 4.98
	*p*	**<0.001**	**0.006**	0.477	**0.019**	**0.013**	0.068	**<0.001**
Overall health rating	Poor	4.41 ± 1.57	2.49 ± 1.46	1.46 ± 1.62	1.00 ± 0.67	1.70 ± 1.41	1.89 ± 0.99	13.14 ± 5.19
	Fair	4.40 ± 1.71	2.43 ± 1.88	1.14 ± 1.14	1.03 ± 0.69	1.54 ± 1.19	1.89 ± 0.96	12.69 ± 5.00
	Good	3.91 ± 1.83	1.86 ± 1.66	1.08 ± 1.38	1.29 ± 0.86	0.96 ± 1.11	1.60 ± 0.93	10.44 ± 5.16
	Excellent	3.07 ± 2.01	1.29 ± 1.54	0.64 ± 1.00	1.19 ± 0.81	0.86 ± 1.09	2.07 ± 1.14	8.93 ± 5.09
	*p*	0.093	**0.022**	0.199	0.473	**0.002**	0.113	**0.006**
EASI ^a^	Mild	2.94 ± 1.73	1.37 ± 1.56	0.79 ± 1.14	0.95 ± 0.77	0.65 ± 0.83	1.44 ± 0.95	7.97 ± 4.53
	Moderate	4.65 ± 1.58	2.29 ± 1.65	1.18 ± 1.37	1.13 ± 0.68	1.41 ± 1.27	1.80 ± 0.96	10.87 ± 5.07
	Severe	4.50 ± 1.55	2.60 ± 1.64	1.58 ± 1.57	1.30 ± 0.82	1.68 ± 1.34	2.13 ± 0.91	14.09 ± 4.67
	*p*	**<0.001**	**<0.001**	**0.012**	0.118	**<0.001**	**0.002**	**<0.001**

Values of domain-specific and overall DLQI expressed as mean ± SD. DLQI: Dermatology Life Quality Index; SD: standard deviation; AD: atopic dermatitis; EASI: Eczema Area and Severity Index; *p*-value calculated using the Mann–Whitney U-test or Kruskal–Wallis test; values in bold are significant. ^a^ EASI categorized as: scores <9 (mild), 9–15 (moderate), and >15 (severe).

**Table 6:** Multiple linear regression models for estimating factors influencing domain-specific DLQI.

Variable		Symptoms and Feelings	Daily Activities	Leisure	Work and School	Personal Relationships	Treatment
		Coef B (95 % CI)	Coef B (95 % CI)	Coef B 95 % CI)	Coef B (95 % CI)	Coef B (95 % CI)	Coef B (95 % CI)
Marital status	Single ^ref^						
	Married	NI	0.25 (−0.24; 0.76)	0.26 (−0.13; 0.66)	NI	0.12 (−0.39; 0.65)	NI
Family history of AD	No ^ref^						
	Yes	0.49 (−0.01; 1.01)	0.30 (−0.19; 0.80)	0.50 (0.11; 0.93) *	NI	0.13 (−0.23; 0.49)	NI
Self-reported global AD severity	Mild ^ref^						
	Moderate	0.77 (−0.11;1.65)	0.16 (−0.69; 1.01)	NI	0.20 (−0.17; 0.57)	0.03 (−0.57; 0.64)	NI
	Severe	1.20 (0.38; 2.02) **	0.56 (−0.22; 1.36)	NI	0.42 (0.07; 0.76) *	0.02 (−0.57; 0.62)	NI
Overall health rating	Excellent ^ref^						
	Good	0.69 (−0.26; 1.64)	0.44 (−0.47; 1.37)	NI	NI	−0.08 (−0.75; 0.58)	NI
	Fair	0.58 (−0.51; 1.67)	0.56 (−0.49; 1.62)	NI	NI	0.18 (−0.58; 0.95)	NI
	Poor	0.68 (−0.39; 1.77)	0.71 (−0.33; 1.76)	NI	NI	0.42 (−0.34; 1.19)	NI
EASI ^a^	Mild ^ref^						
	Moderate	0.87 (0.16; 1.59) *	0.21 (−0.47; 0.91)	0.03 (−0.49; 0.57)	NI	0.53 (0.05; 1.02) *	0.36 (0.05; 0.68) *
	Severe	1.33 (0.50; 2.16) **	0.83 (0.03; 1.64) *	0.58 (0.05; 1.18) *	NI	0.74 (0.17; 1.30) *	0.69 (0.31; 1.06) *

DLQI: Dermatology Life Quality Index; Coef B: unstandardized regression coefficient B; CI: confidence interval; ^ref^: used as reference; NI: not included as not statistically significant at the bivariate level; AD: atopic dermatitis; EASI: Eczema Area and Severity Index; ** *p* < 0.01; * *p* < 0.05. ^a^ EASI categorized as: scores <9 (mild), 9–15 (moderate), and >15 (severe).

**Table 7:** Multiple linear regression model for estimating factors influencing the overall DLQI.

Variable		Unstandardized Coefficients		95% Confidence Interval		*p*
		B	SE	Lower Limit	Upper Limit	
Marital status	Single ^ref^					
	Married	1.25	1.07	−0.87	3.38	0.245
Family history of AD	No ^ref^					
	Yes	1.49	0.74	0.02	2.97	**0.047**
Self-reported global AD severity	Mild ^ref^					
	Moderate	1.47	1.28	−1.06	4.01	0.252
	Severe	2.72	1.18	0.38	5.05	**0.022**
Overall health rating	Excellent ^ref^					
	Good	0.98	1.39	−1.76	3.73	0.481
	Fair	1.20	1.59	−1.93	4.35	0.450
	Poor	2.02	1.59	−1.12	5.16	0.206
EASI ^a^	Mild ^ref^					
	Moderate	1.96	0.99	0.08	3.92	**0.049**
	Severe	4.35	1.20	1.98	6.72	**<0.001**
Constant		4.56	1.79	1.18	7.93	**<0.001**

DLQI: Dermatology Life Quality Index; ^ref^: used as reference; AD: atopic dermatitis; EASI: Eczema Area and Severity Index; B: unstandardized regression coefficient B; SE: standard error; values in bold are significant. ^a^ EASI categorized as: scores <9 (mild), 9–15 (moderate), and >15 (severe).

## Data Availability

All data analyzed during this study are included in this article.

## Supplementary Materials

The following are available online at https://www.mdpi.com/article/10.3390/ijerph182111593/s1, Figure S1: Distribution of patients’ responses on the DLQI items (*n* = 184).

## Abbreviations

AD	atopic dermatitis
CI	confidence interval
DLQI	Dermatology Life Quality Index
EASI	Eczema Area and Severity Index
HRQoL	health-related quality of life
HICs	high-income countries
LMICs	low- and middle-income countries
QoL	quality of life
UKWP	UK Working Party
VIF	variance inflation factor

## References

[1] Langan S.M., Irvine A.D., Weidinger S. (2020). Atopic dermatitis. Lancet.

[2] Luger T., Amagai M., Dreno B., Dagnelie M.A., Liao W., Kabashima K., Schikowski T., Proksch E., Elias P.M., Simon M. (2021). Atopic dermatitis: Role of the skin barrier, environment, microbiome, and therapeutic agents. J. Dermatol. Sci..

[3] Lee H.H., Patel K.R., Singam V., Rastogi S., Silverberg J.I. (2019). A systematic review and meta-analysis of the prevalence and phenotype of adult-onset atopic dermatitis. J. Am. Acad. Dermatol..

[4] Reed B., Blaiss M.S. (2018). The burden of atopic dermatitis. Allergy Asthma Proc..

[5] Ali F., Vyas J., Finlay A.Y. (2020). Counting the Burden: Atopic Dermatitis and Health-related Quality of Life. Acta Derm. Venereol..

[6] Laughter M.R., Maymone M.B.C., Mashayekhi S., Arents B.W.M., Karimkhani C., Langan S.M., Dellavalle R.P., Flohr C. (2021). The global burden of atopic dermatitis: Lessons from the Global Burden of Disease Study 1990–2017. Br. J. Dermatol..

[7] Birdi G., Cooke R., Knibb R.C. (2020). Impact of atopic dermatitis on quality of life in adults: A systematic review and meta-analysis. Int. J. Dermatol..

[8] Blome C., Radtke M.A., Eissing L., Augustin M. (2016). Quality of Life in Patients with Atopic Dermatitis: Disease Burden, Measurement, and Treatment Benefit. Am. J. Clin. Dermatol..

[9] Halioua B., Beumont M.G., Lunel F. (2000). Quality of life in dermatology. Int. J. Dermatol..

[10] Jeon C., Yan D., Nakamura M., Sekhon S., Bhutani T., Berger T., Liao W. (2017). Frequency and Management of Sleep Disturbance in Adults with Atopic Dermatitis: A Systematic Review. Dermatol. Ther. Heidelb..

[11] Tsai T.F., Rajagopalan M., Chu C.Y., Encarnacion L., Gerber R.A., Santos-Estrella P., Llamado L.J.Q., Tallman A.M. (2019). Burden of atopic dermatitis in Asia. J. Dermatol..

[12] Lopez Carrera Y.I., Al Hammadi A., Huang Y.H., Llamado L.J., Mahgoub E., Tallman A.M. (2019). Epidemiology, Diagnosis, and Treatment of Atopic Dermatitis in the Developing Countries of Asia, Africa, Latin America, and the Middle East: A Review. Dermatol. Ther. Heidelb..

[13] Nutten S. (2015). Atopic dermatitis: Global epidemiology and risk factors. Ann. Nutr. Metab..

[14] Pedersen C.J., Uddin M.J., Saha S.K., Darmstadt G.L. (2020). Prevalence of atopic dermatitis, asthma and rhinitis from infancy through adulthood in rural Bangladesh: A population-based, cross-sectional survey. BMJ Open.

[15] Pedersen C.J., Uddin M.J., Saha S.K., Darmstadt G.L. (2021). Prevalence and psychosocial impact of atopic dermatitis in Bangladeshi children and families. PLoS ONE.

[16] Basra M.K.A., Fenech R., Gatt R.M., Salek M.S., Finlay A.Y. (2008). The Dermatology Life Quality Index 1994–2007: A comprehensive review of validation data and clinical results. Br. J. Dermatol..

[17] Williams H.C., Burney P.G., Hay R.J., Archer C.B., Shipley M.J., Hunter J.J., Bingham E.A., Finlay A.Y., Pembroke A.C., Graham-Brown R.A. (1994). The U.K. Working Party’s Diagnostic Criteria for Atopic Dermatitis. I. Derivation of a minimum set of discriminators for atopic dermatitis. Br. J. Dermatol..

[18] Hanifin J.M., Thurston M., Omoto M., Cherill R., Tofte S.J., Graeber M. (2001). The eczema area and severity index (EASI): Assessment of reliability in atopic dermatitis. EASI Evaluator Group. Exp. Dermatol..

[19] Finlay A.Y., Khan G.K. (1994). Dermatology Life Quality Index (DLQI)—A simple practical measure for routine clinical use. Clin. Exp. Dermatol..

[20] Hongbo Y., Thomas C.L., Harrison M.A., Salek M.S., Finlay A.Y. (2005). Translating the science of quality of life into practice: What do dermatology life quality index scores mean?. J. Investig. Dermatol..

[21] Vakharia P.P., Chopra R., Sacotte R., Patel N., Immaneni S., White T., Kantor R., Hsu D.Y., Silverberg J.I. (2018). Validation of patient-reported global severity of atopic dermatitis in adults. Allergy.

[22] Faul F., Erdfelder E., Buchner A., Lang A.G. (2009). Statistical power analyses using G*Power 3.1: Tests for correlation and regression analyses. Behav. Res. Methods.

[23] Majbauddin A., Otani S., Tsunekawa A., Haregeweyn N., Abeje M.T., Nigussie Z., Alam I., Qing Q., Masumoto T., Kurozawa Y. (2020). The Influence of Income and Livelihood Diversification on Health-Related Quality of Life in Rural Ethiopia. Int. J. Environ. Res. Public Health.

[24] Holm J.G., Agner T., Clausen M.L., Thomsen S.F. (2016). Quality of life and disease severity in patients with atopic dermatitis. J. Eur. Acad. Dermatol. Venereol..

[25] Sanchez-Perez J., Dauden-Tello E., Mora A.M., Lara Surinyac N. (2013). Impact of atopic dermatitis on health-related quality of life in Spanish children and adults: The PSEDA study. Actas Dermosifiliogr..

[26] Silverberg J.I., Gelfand J.M., Margolis D.J., Boguniewicz M., Fonacier L., Grayson M.H., Simpson E.L., Ong P.Y., Chiesa Fuxench Z.C. (2018). Patient burden and quality of life in atopic dermatitis in US adults: A population-based cross-sectional study. Ann. Allergy Asthma Immunol..

[27] Huang J., Choo Y.J., Smith H.E., Apfelbacher C. (2021). Quality of life in atopic dermatitis in Asian countries: A systematic review. Arch. Dermatol. Res..

[28] Coghi S., Bortoletto M.C., Sampaio S.A., Andrade Junior H.F., Aoki V. (2007). Quality of life is severely compromised in adult patients with atopic dermatitis in Brazil, especially due to mental components. Clin. Sao Paulo.

[29] Maksimovic N., Jankovic S., Marinkovic J., Sekulovic L.K., Zivkovic Z., Spiric V.T. (2012). Health-related quality of life in patients with atopic dermatitis. J. Dermatol..

[30] Holm E.A., Wulf H.C., Stegmann H., Jemec G.B.E. (2006). Life quality assessment among patients with atopic eczema. Br. J. Dermatol..

[31] Hsieh B.J., Shen D., Hsu C.J., Chan T.C., Cho Y.T., Tang C.H., Chu C.Y. (2021). The impact of atopic dermatitis on health-related quality of life in Taiwan. J. Formos. Med. Assoc..

