# Supplementary figures and images for: The Influence of Atopic Dermatitis on Health-Related Quality of Life in Bangladesh
Source: Int J Environ Res Public Health. 2021 Nov 4;18(21):11593. doi: 10.3390/ijerph182111593 (PMC8583474; doi:10.3390/ijerph182111593)

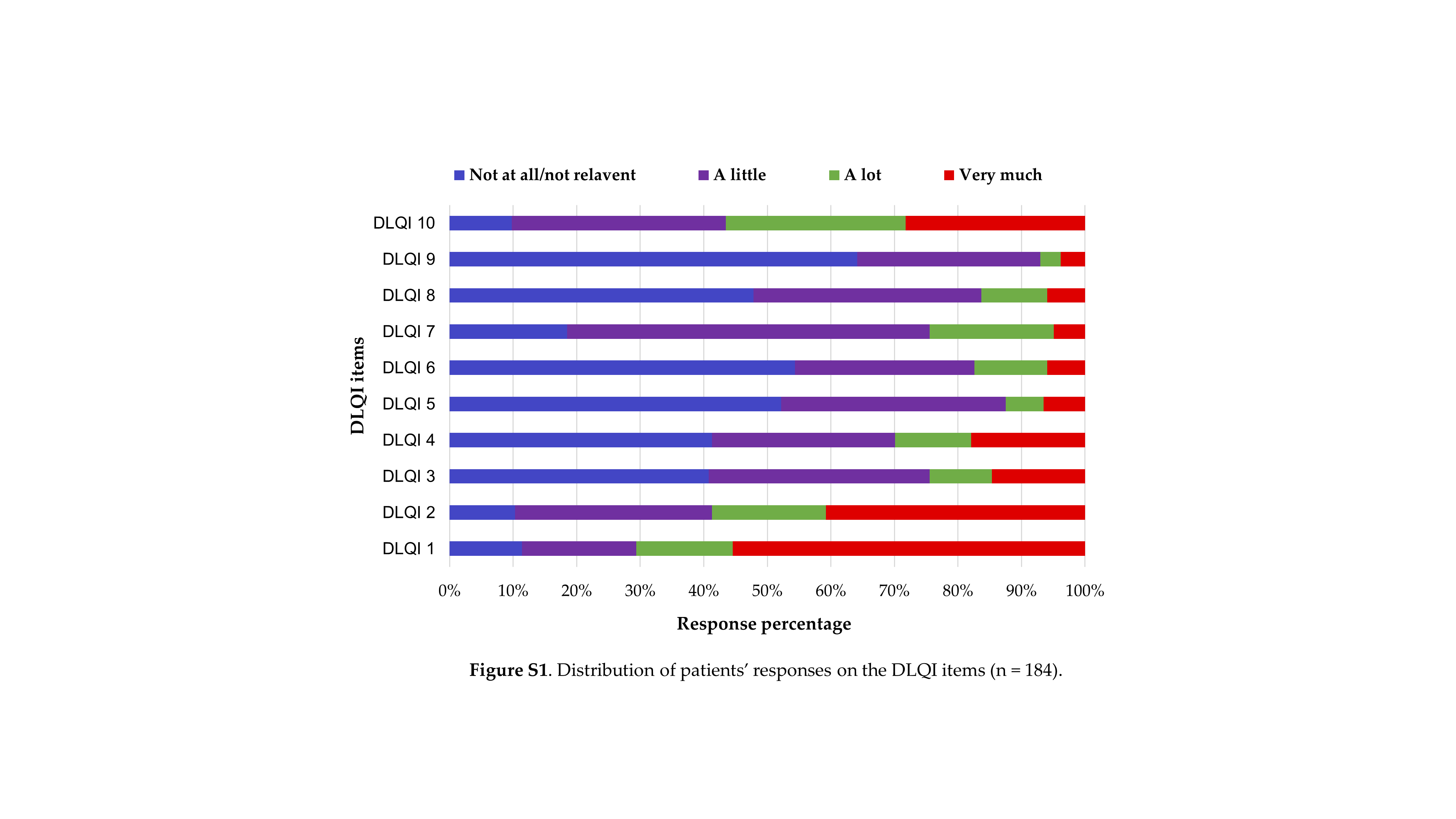

Supplement: Supplementary file 1 [file ijerph-18-11593-s001.zip › Figure S1.tif]
